# Optimization of the Frying Process for Maximizing Crispiness of Scallop (*Patinopecten yessoensis)* Adductor Muscle Snacks Using Vacuum Low-Temperature Frying

**DOI:** 10.3390/foods13244091

**Published:** 2024-12-17

**Authors:** Sun-Young Park, Sang-In Kang, Jin Kim, Young-Hyun An, Ga-Bin Lee, Si-Hyeong Park, Jung-Suck Lee

**Affiliations:** Department of Seafood Science and Technology, Institute of Marine Industry, Gyeongsang National University, 2-9, Tongyeonghaean-ro, Tonyeong-si 53064, Gyeongsangnam-do, Republic of Korea; tjsdud3591@gnu.ac.kr (S.-Y.P.); sikang@gnu.ac.kr (S.-I.K.); kimjin501@gnu.ac.kr (J.K.); younghyun@gnu.ac.kr (Y.-H.A.); gabin1010@gnu.ac.kr (G.-B.L.); sipark@gnu.ac.kr (S.-H.P.)

**Keywords:** scallops, *Patinopecten yessoensis*, vacuum low-temperature frying, crispy snack, optimized, temperature, time

## Abstract

Scallops, an economically important seafood, are popular as fried snacks. Vacuum low-temperature frying creates crispy, healthier foods that meet consumer demand for nutritious snacks with excellent texture. However, research on vacuum frying for shellfish products remains limited. This study aimed to optimize the process for developing a healthy, crispy snack that retains the original shape of the Yesso scallop (*Patinopecten yessoensis*) adductor muscle using vacuum low-temperature frying technology. The effects of various frying methods on the scallops were analyzed. The vacuum low-temperature frying process was optimized based on evaluations of physical, chemical, and sensory properties. Frying conditions were optimized using response surface methodology, with temperature (X1: 90.1–109.9 °C) and time (X2: 186–774 s) as variables. Based on moisture (5.6 ± 0.1 g/100 g), hardness (1470 ± 5.0 g/cm^2^), and sensory chewiness (7.6 ± 0.2 points) analyses, 99.9 °C and 480 s were identified as the optimal conditions. Validation was conducted through sensory evaluation by 30 trained panelists. Scallops produced under these optimal frying conditions exhibited low water activity (aw) (0.46), desirable texture (1428 g/cm^2^), palatability (7.9 points), and high protein content (45.6 g/100 g). The predicted and experimental values for frying temperature and time showed strong agreement, validating the reliability of the optimization model.

## 1. Introduction

Scallops are among the most popular seafood items worldwide, known for their unique taste and texture, with approximately 400 species identified to date [1]. They are an economically valuable marine resource, offering higher protein content than most other shellfish. Rich in amino acids, including lysine, threonine, and taurine, scallops are particularly beneficial for supporting growth and development in children. They also contain various flavor components, such as glycogen, glycine, and succinic acid [2].

In 2020, the total global production of scallops was 2,742,404 M/T, with China leading at 1,746,238 M/T (63.7%), followed by Japan (497,000 M/T; 18.1%), and the USA (185,256 M/T; 6.8%). The Republic of Korea ranked 15th with 5722 M/T. Notably, Asia dominates production, with China, Japan, the Republic of Korea, and Thailand (4785 M/T, 0.2%) accounting for 82.2% of global production. In Asia, scallops are primarily consumed grilled, steamed, raw, or in soup, with fresh or refrigerated forms comprising over 95% of total consumption [3,4]. To ensure a stable supply of scallops, there has been a shift from raw materials to processed products, highlighting the need for diversified processing methods [5].

Research on Yesso scallops (*Patinopecten yessoensis*) has focused on their ecological characteristics [6], amino acid composition in different tissues [4], nutritional components related to habitat and cultivation methods [7], quality [5], and immune responses [8]. Moreover, most previous studies have focused on aquaculture or nutritional characteristics, with only one study evaluating the production and quality characteristics of high-value canned products using domestic *P. yessoensis* [9]. Therefore, research on product development for *P. yessoensis* is limited.

Recently, the food market has shifted from home meal replacements to “snackification”. This trend reflects a shift in eating habits, particularly among millennials, from the traditional three meals a day to portable, convenient snacks that can be consumed in short periods, such as during travel or breaks. This trend is growing rapidly, with snacks increasingly being used as meal replacements. Consequently, there is a growing demand for healthy, high-quality snacks made from nutrient-rich ingredients, such as scallop adductor muscles, which offer both health benefits and superior quality compared to traditional snack options. Key processing technologies used in snack manufacturing include extrusion [10], rolling [11], deep frying [12], drying [13], and air frying [14]. With the increasing focus on health, convenience, and quality, hot air frying and vacuum low-temperature frying have emerged as popular alternatives to traditional atmospheric deep frying. Atmospheric deep frying involves short-term heat treatment at high temperatures (160–180 °C) for 2–3 min, causing significant microstructural changes on the surface and interior of the food [15]. Although this method enhances crispiness and imparts a unique color, flavor, and texture, high-temperature frying leads to quality degradation due to the breakdown of tocopherols, essential amino acids, and fatty acids [16]. Air frying is a method in which a fan inside the air fryer circulates hot air, which is drawn from outside, creating dry, hot air that fries the food [17,18,19]. This process rapidly removes moisture, making the food crispy while expelling excess oil. Although air frying results in lower-fat, lower-calorie products than those obtained by atmospheric deep frying, oil coating may still be required for foods with a low-fat content [20,21,22]. This method has additional limitations such as the formation of acrylamide for starchy foods, which can pose health risks, and high energy consumption [23].

Vacuum low-temperature frying is a recently developed method in which oil serves as a heating medium. Under vacuum conditions, moisture in the raw material vaporizes, allowing drying and frying to occur simultaneously [24]. The principle is similar to boiling in water at temperatures below 100 °C when pressure is reduced below atmospheric levels [25,26]. Vacuum frying can be performed at lower temperatures than atmospheric frying with similar effects. Foods fried using this method benefit from significantly reduced oil oxidation and contain lower levels of harmful compounds such as trans fats, polar compounds, and acrylamide [27,28,29,30,31]. With the increasing number of health-conscious consumers, there is a growing demand for low-sugar, low-calorie snack products that retain the original shape of the raw material. Notably, vacuum frying can effectively meet the needs of consumers seeking healthy foods with excellent texture. Studies using vacuum low-temperature frying include the development of fishballs [32], pineapple snacks [33], mango chips [34], mushroom chips [35], and cassava chips [36]. However, most of these studies have focused on agricultural products, with limited research on the production of nutritionally superior processed shellfish products using vacuum frying.

The aim of this study was to optimize the frying process to develop a healthy and crispy snack from Yesso scallop adductor muscle while preserving its original shape. Vacuum low-temperature frying technology was employed for this purpose, and its effectiveness was compared with atmospheric frying and air frying in terms of nutritional characteristics.

## 2. Materials and Methods

### 2.1. Materials

The Yesso scallops (*Patinopecten yessoensis*) used in this experiment were sourced from the Shrimp Mall in April 2023. A total of 2000 g of frozen adductor muscles with a diameter ranging from 36.1 to 38.4 mm (average 37.1 ± 0.9 mm), height from 9.9 to 10.1 mm (average 10.0 ± 0.1 mm), and weight from 11.7 to 12.5 g (average 12.2 ± 0.3 g) were utilized. Additionally, the supplementary ingredients for manufacturing the scallop snacks, including high fructose syrup (Daesang Co., Ltd., Seoul, Republic of Korea), refined salt (Hanju Co., Ltd., Ulsan, Republic of Korea), and soybean oil (Daesang Co., Ltd., Seoul, Republic of Korea), were obtained from online shopping malls in April 2022. All reagents used in the experiments were of analytical grade.

### 2.2. Manufacturing Method of Yesso Scallop Adductor Muscle Snacks Using Vacuum Frying, Air Frying and Atmospheric Frying

To compare the quality of scallop snacks prepared using vacuum frying technology, two control groups (scallop snacks processed by atmospheric frying and air frying) were prepared as follows. The control samples used scallops treated under the same pre-processing conditions as those for the vacuum-fried snacks. The Yesso scallops were thawed, washed, and dehydrated, then sliced to a height of 2.5 mm. For the sugar-salt soaking, the scallops were immersed in a solution [79.0% water (*v*/*v*), 19.7% high fructose (*w*/*v*), and 1.3% salt (*w*/*v*)] for 30 min, then dehydrated and frozen at −18 °C for at least 3 h. Lastly, (1) vacuum-fried snacks were fried in a vacuum fryer (BT-1E, Kiyomoto Co., Ltd., Nobeoka, Japan) for 186–774 s, (2) atmospheric-fried snacks were prepared using a two-step frying process in an electric fryer (FR-3220KR; Tefal, Sarcelles, France) filled with soybean oil, frying first at 140 °C for 2 min, followed by a second fry at 170 °C for 1 min, (3) air-fried snacks were cooked in an air fryer (AO-16LS; Inic Corp., Anyang, Republic of Korea) at 170 °C for 6 min, with approximately 2 mL of soybean oil applied per piece to improve texture.

### 2.3. Proximate Components and Water Activity

To determine its proximate components, the Yesso scallop was analyzed using the atmospheric pressure drying method for moisture, the semi-micro Kjeldahl method for crude protein, the Soxhlet method for crude fat, and the dry ashing method for ash, according to the AOAC [37]. The carbohydrate content was calculated as 100 − (moisture content + protein content + crude fat content + ash content).

Water activity was measured using 3 g of the ground sample with a Thermoconstanter (Climmate-set-aw, Novasina AG, Schwyz, Switzerland).

### 2.4. Hardness

Texture was expressed as fracturability obtained from a compression test, slightly modified from the method described by Salvador et al. [38].

The texture of the Yesso scallop adductor muscle snack was measured by placing the snack on an empty cylinder and compressing it using a texture analyzer (CT3-1000; Brookfield, Middleboro, MA, USA) equipped with a 50 mm spherical plunger (TA50 cylindrical probe). The probe’s trigger load was set to 5 g, the test speed to 1.0 mm/s, and the sample deformation rate to 50%.

### 2.5. Scanning Electron Microscope (SEM)

The microstructure of the scallop adductor muscle snack was observed using a scanning electron microscope as described by Rattanasatheirn et al. [39]. After cutting, the scallop snacks prepared by atmospheric pressure frying, air frying, and vacuum low-temperature frying were immersed in ether (Daejung Chemicals & Metal Co., Ltd., Siheung, Republic of Korea) for 4 h to completely remove all surface oil. The samples were shaped into a regular shape (4 mm × 4 mm × 4 mm), then immersed in 2.5% glutaraldehyde with 0.2 M phosphate buffer (pH 7.2; as a solvent) at 4 ± 1 °C for 2 h. Thereafter, these samples were washed thrice with deionized water for 15 min and then dehydrated for 30 min each in 20, 40, 60, 80, 100% ethanol for a total of 150 min. These dehydrated scallop snacks prepared by atmospheric pressure frying, air frying, and vacuum frying were dried (at 10 °C for 10 min, then at 32 °C for 10 min) using a critical point dryer (13200-AB, SPI SUPPLIES, West Chester, PA, USA). The dried scallop snacks prepared by atmospheric pressure frying, air frying, and vacuum frying were coated with 100% gold (nanopowder, 200 nm particle size (SEM), 99.9% (metals basis), Sigma-Aldrich Co., Ltd., St. Louis, MO, USA) using an ion sputter coater (Sputter Coater SPT-20, Coxem Co., Republic of Korea). The microstructure of the gold-coated scallop adductor muscle snack was observed at 500× magnification using a scanning electron microscope (SEM; JSM-7610F; Jeol Ltd., Tokyo, Japan).

### 2.6. Sensory Evaluation

After obtaining approval (No. GIRB-G22-Y-0070) for research using human subjects from the Institutional Review Board (IRB; Jinju, Republic of Korea) of Gyeongsang National University (Jinju, Republic of Korea) in accordance with the Enforcement Decree of Bioethics and Safety Act, a sensory evaluation was conducted on the scallop adductor muscle snack prototype. The preference evaluation was conducted immediately after the preparation of the snacks using a 9-point hedonic scale, where 1 indicated “dislike extremely” and 9 indicated “like extremely”. Thirty participants (15 males and 15 females; age distribution, 20–45 years; average age, 30 years) who are students at Gyeongsang National University, Tongyeong, Republic of Korea, assessed the taste, texture, color, smell, and overall acceptability of the prototype. This evaluation was performed in accordance with the methodology outlined by Lawless and Heymann [40].

### 2.7. Establishment of Scallop Adductor Muscle Snack Conditions by Response Surface Methodology (RSM)

A central composite design (CCD) was performed by utilizing the response surface method (RSM) to optimize the vacuum low-temperature hot-water process in manufacturing the Yesso scallop snack (Table 1). As experimental variables, the vacuum low-temperature hot-water bath temperature (X1, 90.1–109.9 °C) and time (X2, 186–774 s) determined through the preliminary experiment were set as independent variables, and 11 sample spheres were prepared and tested by encoding them in five steps according to the central synthesis plan. In this case, the dependent variables were set as moisture (Y1), hardness (Y2), and functional chewability (Y3) (Table 2).

In addition, the prediction and confirmation of the optimum point were conducted using response surface regression (RSREG) analysis with the MINITAB Statistics Program (MINITAB Ver. 18, Minitab, State College, PA, USA) 

### 2.8. Acid Value

The acid value of scallop adductor muscle snacks, prepared using various frying methods, was measured after storing the samples at room temperature for 28 days. The samples were analyzed at 7-day intervals during the storage period. The measurement followed the AOCS method [41] and was expressed as milligrams of KOH per 1 mL of 0.1 N-KOH. Approximately 1 g of sample oil was mixed with 40 mL of ether (2:1, *v*/*v*) in an Erlenmeyer flask. After adding 2–3 drops of 1% phenolphthalein, the mixture was shaken until the oil dissolved. The acid value was determined by titrating with 0.1 N-KOH ethanol solution, with the endpoint being a faint pink color lasting 30 s, after which the acid value was calculated.

$$Acid\;value\;(mg/g)\;=\;\frac{5.611\times [(Volume\;of\;titrant\;for\;sample,\;mL)\;-\;(Volume\;of\;titrant\;for\;blank)]\;\times \;N}{The\;amount\;(g)\;of\;the\;sample}$$

### 2.9. Peroxide Value

The peroxide value of scallop adductor muscle snacks, prepared using various frying methods, was measured after storing the samples at room temperature for 28 days. The samples were analyzed at 7-day intervals during the storage period. The extracted sample oil was analyzed following the AOCS [41]. For preparation, 0.5–1.0 g of the sample oil was placed in an Erlenmeyer flask, followed by 30 mL of acetic acid–chloroform mixture (1:1, *v*/*v*) and 1 mL of saturated KI solution. The mixture was flushed with nitrogen gas and shaken well. The peroxide value was determined by adding 1% starch solution as an indicator and titrating with 0.01N Na_2_S_2_O_3_ solution, and the value was calculated accordingly.

$$Peroxide\;value\;=\;\frac{[(Volume\;of\;titrant\;for\;sample,\;mL)\;-\;(Volume\;of\;titrant\;for\;blank)]\;\times \;N\;\times \;10}{Weight\;of\;the\;sample,\;g}$$

### 2.10. Statistical Analysis

For the analysis of standard deviation and determination of significant differences at the 5% level, ANOVA was performed using SPSS statistical software (SPSS for Windows, version 10.1). Following the analysis of variance, Duncan’s multiple range test was applied to further evaluate specific group differences.

## 3. Results and Discussion

### 3.1. Changes in General Composition Under Various Frying Methods

Heat treatment can induce changes in general composition through physical and chemical decomposition, and moisture and fat contents are essential parameters in vacuum frying [35]. In this context, the general composition and energy content under various frying methods are presented in Table 3. The moisture content per 100 g of scallop snacks was highest in C-AF at 36.0 g, followed closely by C-DF at 35.5 g, while P-VF showed a significantly lower value of 5.9 g (*p* < 0.05). These results indicate that the vacuum frying method induces microstructural changes while simultaneously facilitating heating and drying under reduced pressure, thus influencing moisture release [35].

The crude protein content per 100 g of scallop snacks was significantly higher for P-VF at 45.6 g than for C-AF at 33.9 g and C-DF at 27.9 g (*p* < 0.05). The ash content per 100 g was also significantly higher for P-VF at 6.5 g than for C-AF at 2.9 g and C-DF at 2.8 g (*p* < 0.05). The higher crude protein and ash contents in vacuum-fried scallop snacks can be attributed to their lower moisture contents. The crude fat content per 100 g was significantly higher for C-DF at 12.2 g than for C-AF at 10.9 g and P-VF at 8.8 g. The lower fat content observed in vacuum frying compared with conventional frying methods is due to the oil removal process during vacuum frying, which reduces oil absorption both on the surface and within the product. In contrast, air frying involves only a small amount of oil, leading to a significantly lower fat content than both conventional and vacuum frying methods. The energy content per 100 g of scallop snacks was highest for P-VF at 402.6 kcal, followed by C-DF at 312.8 kcal and C-AF at 290.7 kcal. The higher caloric content in P-VF can be attributed to its lower moisture content and significantly higher protein content.

### 3.2. Water Activity and Texture Under Various Frying Methods

Water activity (a_w_) was evaluated to predict changes in moisture in multi-component snack foods and to explain how moisture-related quality attributes, such as texture after pretreatment and frying processes, are affected [36].

Fracturability refers to the resistance of a snack to breaking under physical force (i.e., the force applied until it initially fractures). This parameter is generally high in foods with a low moisture content and low elasticity, such as cookies or crackers. In contrast, foods with a high moisture content, such as bread or cake, possess elasticity and thus have relatively low fracturability [42].

**Figure 1:**
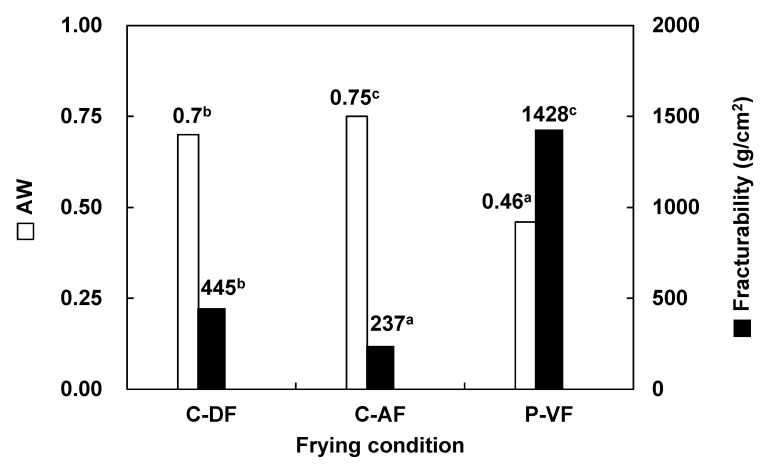
Water activity and crispiness of scallop adductor muscle snacks by frying condition. C-DF, deep frying; C-AF, air frying; P-VF, vacuum low-temperature frying. The letters a–c in the data indicates a significant difference at *p* > 0.05.

Water activity and fracturability under various frying methods are shown in Figure 1. The water activity was highest in C-AF at 0.75, followed by C-DF snacks at 0.70 and P-VF snacks at 0.46. In terms of fracturability, the vacuum-fried snacks had the highest value at 1428 g/cm^2^, followed by atmospheric fried snacks at 445 g/cm^2^ and air-fried snacks at 237 g/cm^2^. These results indicate that a lower water activity is associated with a higher fracturability.

In the vacuum state, the boiling point of oil is lowered, which promotes the evaporation of moisture within the food and simultaneously increases crispiness. As a result, the rate of dehydration is quicker for P-VF snacks than for other types [36].

### 3.3. Microstructural Analysis Under Various Frying Methods

**Figure 2:**
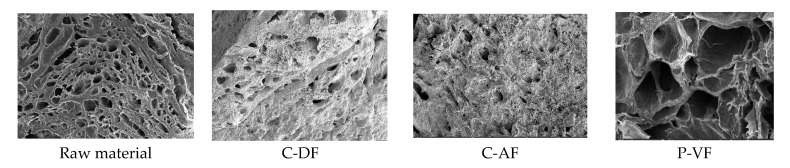
Scanning electron microscope of scallop adductor muscle snacks by frying conditions. C-DF, deep frying; C-AF, air frying; P-VF, vacuum low-temperature frying.

To examine structural changes in scallop adductor muscle snacks, the microstructures of raw scallops and three types of scallop adductor muscle snacks prepared using different frying methods were compared, as shown in Figure 2.

Compared with raw scallop adductor snacks, the three types of scallop adductor snacks that were pre-treated with a sugar–salt immersion solution exhibited a denser structure. This may be due to the viscosity of the sugar filling the spaces between the muscle fibers, thereby reducing the overall porosity. Additionally, the surfaces of the three fried products (C-DF, C-AF, and P-VF) exhibited structural changes in the internal microcells, consistent with previous findings [43,44]. The C-DF snacks exhibited irregular holes, while the C-AF snacks had relatively regular-shaped holes. In contrast, P-VF snacks had larger and more irregular holes compared with those in the other groups. The C-AF snacks, which experienced a slower rate of heat transfer compared with C-DF and P-VF, had a lower moisture removal rate, resulting in relatively regular and smaller holes. In the case of P-VF snacks, the rapid evaporation of moisture contained in the raw materials is believed to have led to the rapid generation and removal of steam, resulting in the formation of a porous structure.

Gong et al. [45] observed the microstructure of scallop adductor muscle snacks processed by C-DF and P-VF, confirming that P-VF-treated snacks exhibit greater porosity than that of C-DF snacks. Additionally, Hsieh et al. [46], Math et al. [47], Mallikarjunan [48], Van Koerten et al. [49], and Surojanametakul et al. [50] have shown that the formation of a porous structure in fried foods allows moisture to evaporate and pores to form, contributing to a crispy texture. Therefore, it is believed that P-VF-treated snacks will have a higher crispiness than that of control snacks.

### 3.4. Sensory Evaluation Under Various Frying Methods

The results of a sensory evaluation (preference for texture) conducted by panelists for scallop adductor muscle snacks processed by atmospheric frying, air frying, and vacuum low-temperature frying are presented in Table 4.

With respect to the appearance of the scallop adductor muscle snacks, P-VF received the highest preference score of 8.0, followed by C-DF with 5.9 and C-AF with 4.9. The lower score for C-AF can be attributed to the oil application, which caused burning only on the surface, resulting in a significantly lower score. The scores for aroma showed significant differences among samples (*p* < 0.05).

In terms of texture, the highest score was obtained for P-VF at 8.3, followed by C-DF at 5.7 and C-AF at 5.0 (*p* < 0.05). These results were consistent with the microstructure analyses, revealing that the porous structure in the P-VF snacks allowed moisture to evaporate, resulting in a crispy texture.

### 3.5. Optimization of Vacuum Frying Conditions for Yesso Scallop Adductor Muscle Snacks

#### 3.5.1. Effects of Vacuum Frying Temperature and Time on the Moisture Content

To optimize the vacuum frying conditions (frying temperature and time) for the manufacture of Yesso scallop snacks, 11 sample groups were randomly prepared by encoding X1 (treatment temperature) and X2 (treatment time) in five levels according to the central composite design presented in Table 2.

**Figure 3:**
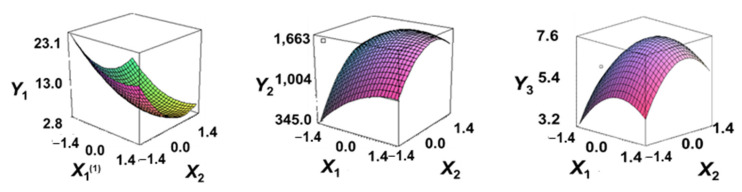
Three-dimensional response surface plots for vacuum frying process optimization of scallop adductor muscle snacks based on *Y*_1_ (moisture, g/100 g), *Y*_2_ (fracturability, g/cm^2^) and *Y*_3_ (sensory chewiness, score). *X*_1_, vacuum frying temp. (°C); *X*_2_, vacuum frying time (s).

The dependent variable moisture (Y1) decreased as the code values of X1 and X2 moved from –1.414 to 0.962 and 0.798, followed by a slight increase (Figure 3). Generally, only significant terms in the quadratic regression equation derived from the RSREG using MINITAB are retained [51].

Therefore, in the RSREG analysis of the dependent variable moisture (Y1), four terms were significant, the linear terms X1 and X2 and the quadratic terms X1^2^ and X2^2^ (*p* < 0.05) (Table 5). The model had a *p*-value of 0.006 for the lack of fit test, a parameter used to evaluate the goodness of fit of the designed model [52], indicating an adequate model. Furthermore, the coefficient of determination for moisture (Y1) was 0.943, and the *p*-value for the model of yield (Y1) was 0.000. These findings indicated that all terms in the designed model were adequate [53].

These results confirmed that the treatment conditions for vacuum low-temperature frying temperature and time affect the moisture content in Yesso scallop snacks significantly.

#### 3.5.2. Effect of Vacuum Low-Temperature Frying Temperature and Time on Hardness

The hardness (Y2) results for the 11 samples manufactured according to the RSM design are presented in Table 2.

Hardness (Y2) increased initially as the code values of X1 and X2 moved from –1.414 to 0.846 and 0.546, followed by a gradual decline (Figure 3).

In the RSREG analysis, the linear, quadratic, and interaction terms for the independent variables temperature and time were significant. In particular, for hardness (Y2), the significant terms included the linear terms X1 and X2, the quadratic terms X1^2^ and X2^2^, and the interaction term X1X2, totaling five significant terms (Table 5).

Additionally, the *p*-value for the lack of fit of the response model equation was 0.012. The coefficient of determination (*R*^2^) for hardness (Y2) was 0.993, and the *p*-value for the model for hardness (Y2) was 0.000, suggesting a good model fit.

The results of this experiment indicated the vacuum low-temperature frying temperature and time have significant effects on the hardness of Yesso scallop snacks.

#### 3.5.3. Effect of Vacuum Low-Temperature Frying Temperature and Time on Sensory Chewiness

The results of sensory chewiness (Y3) analyses for the 11 samples manufactured according to the RSM design are presented in Table 2. Sensory chewiness (Y3) increased sharply as the code values of X1 and X2 moved from −1.414 to 0.300 and 0.300, followed by a decreasing trend thereafter (Figure 3).

In the RSREG analysis, significant linear, quadratic, and interaction terms for sensory chewiness (Y3) included the linear terms X1 and X2, as well as the quadratic terms X1^2^ and X2^2^, totaling four significant terms (Table 5). Furthermore, the *p*-value for the lack of fit test of the response model was 0.023, and the coefficient of determination (*R*^2^) for chewiness (Y2) was 0.893. The *p*-value for the model of chewiness (Y2) was 0.003, confirming the suitability of the model.

The results of this experiment confirmed that the vacuum low-temperature frying temperature and time have significant effects on the sensory chewiness of Yesso scallop snacks.

### 3.6. Optimization of Vacuum Frying Conditions for Scallop Adductor Muscle Snacks

For scallop adductor muscle snacks, it is crucial to maintain appropriate moisture levels and a crispy texture. If the vacuum low-temperature frying temperature and time are set too high, the moisture content may drop excessively, leading to a hard texture that is not in line with consumer preferences. If the vacuum low-temperature frying temperature and time are set too low, there is a risk that the snacks will not cook properly, resulting in a high moisture content and low crispness, with the potential for issues during distribution.

Therefore, the range of values for the vacuum low-temperature frying temperature (X1) and time (X2) based on consumer preferences for scallop snacks was established. The ranges were set at 4.2 to 18.3 g/100 g for moisture, 369 to 1581 g/cm^2^ for hardness, and 5.1 to 7.5 points for sensory chewiness. The target values for these variables were determined by referencing the preliminary experimental results, indicating consumer preferences of 5.6 g/100 g for moisture, 1480 g/cm^2^ for hardness, and the maximum value for sensory chewiness.

The optimal conditions (i.e., vacuum low-temperature frying temperature and time) that can simultaneously maximize each dependent variable for scallop snacks were determined using MINITAB. The optimal processes with respect to the independent variables are presented in Table 6. Considering the target values for the independent variables (X1 and X2) for moisture (Y1), the optimal coded values were 0.00 and 1.41, which corresponded to actual values of 100.0 °C and 777 s, respectively. For hardness (Y2), the optimal coded values were both 0.00, corresponding to actual values of 100.0 °C and 480 s. For sensory chewiness (Y3), the optimal coded values were both 0.30, which corresponded to actual values of 102.1 °C and 543 s.

The coded values of the independent variables (X1 and X2) that simultaneously met all vacuum low-temperature frying conditions for scallop snacks were −0.01 and 0.00, corresponding to actual values of 99.9 °C and 480 s, respectively. The predicted dependent variables for the vacuum low-temperature frying scallop snacks produced under these optimal conditions (frying temperature of 99.9 °C and frying time of 480 s) were 5.7 g/100 g for moisture, 1478 g/cm^2^ for hardness, and 7.5 points for sensory chewiness (Table 6).

The actual dependent variables for the scallop snacks produced under these optimal conditions were 5.6 ± 0.1 g/100 g for moisture, 1470 ± 5.0 g/cm^2^ for hardness, and 7.6 ± 0.2 points for sensory chewiness. Based on these results, the proposed response surface analysis model is suitable for determining the optimal frying temperature and time (Table 7).

### 3.7. Changes in Lipid Oxidation During Frying

**Figure 4:**
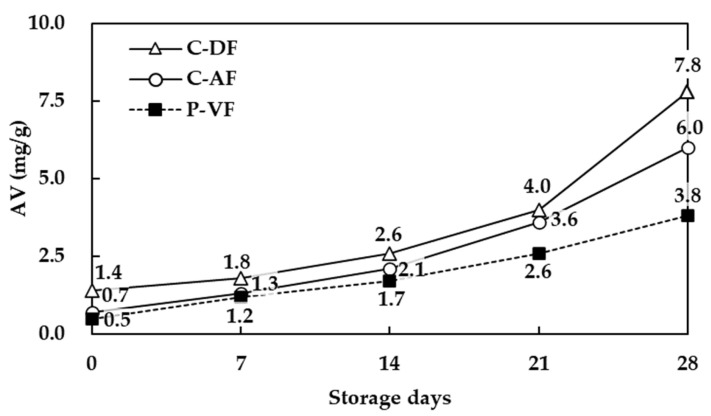
Acid value of scallop adductor muscle snacks by frying technology. C-DF, deep frying; C-AF, air frying; P-VF, vacuum low-temperature frying.

**Figure 5:**
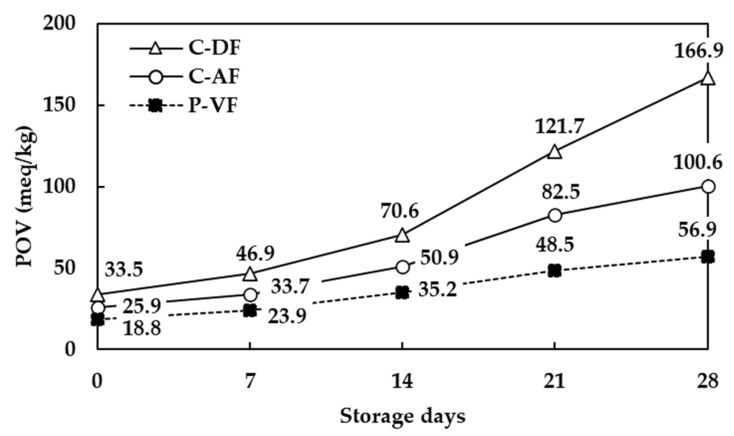
Peroxide value of scallop adductor muscle snacks by frying technology. C-DF, deep frying; C-AF, air frying; P-VF, vacuum low-temperature frying.

The acidity values and peroxide values of scallop adductor muscle snacks prepared with different frying methods and stored for 28 days, determined at 7-day intervals, are shown in Figure 4 and Figure 5.

Acidity and peroxide values are indicators of the degree of rancidity. Acidity is also referred to as free fatty acid value and indicates the content of free fatty acids formed by the hydrolysis of lipid molecules, rather than the inherent characteristics of the fat itself. The peroxide value measures the amount of peroxides, such as hydroperoxides, generated through auto-oxidation. This value tends to increase when fried products are stored for extended periods.

The acidity of scallop adductor muscle snacks tended to increase, regardless of the frying method used. C-DF (regression equation y = 1.5x − 0.1) increased significantly and rapidly, followed by C-AF (regression equation y = 1.3x − 1.1), while P-VF (regression equation y = 0.8x − 0.4) had the slowest rate of increase. Specifically, the acidity values for the Yesso scallop snacks immediately after frying were 1.4 mg/g (C-DF), 0.7 mg/g (C-AF), and 0.5 mg/g (P-VF), and these values continued to rise over time, reaching 7.8 mg/g (C-DF), 6.0 mg/g (C-AF), and 3.8 mg/g (P-VF) on day 28.

Similarly, the peroxide values of scallop adductor muscle snacks exhibited an increasing trend during the storage period, irrespective of the frying method. The C-DF (regression equation y = 34.2x − 14.6) showed a notably rapid increase, followed by C-AF (regression equation y = 19.8x − 0.7), while P-VF (regression equation y = 10.1x + 6.4) had the slowest rate of increase. Thus, the peroxide values for the Yesso scallop snacks immediately after frying were 33.5 meq/kg (C-DF), 25.9 meq/kg (C-AF), and 18.8 meq/kg (P-VF), which continued to rise, reaching 166.9 meq/kg (C-DF), 100.6 meq/kg (C-AF), and 56.9 meq/kg (P-VF) on day 28.

Previous studies, including Pandey [54], Andrés-Bello [55], and Ayustaningwarno [56], have shown that atmospheric frying results in a higher rate of auto-oxidation (due to the use of higher temperatures) than that of vacuum frying, consistent with the results for scallop adductor muscle snacks. It is believed that the presence of fats in products leads to auto-rancidity as they combine with oxygen [57,58].

These results suggest that atmospheric frying, utilizing higher temperatures than those for vacuum frying, leads to faster rates of auto-oxidation, which in turn forms a porous structure during the frying process. In the case of atmospheric frying, where no oil removal process is applied, oil is easily absorbed, and there is a rapid deterioration in quality due to the quick contact between the oil and air, leading to rancidity.

## 4. Conclusions

In this study, response surface methodology was employed to optimize the conditions for vacuum low-temperature frying (P-VF), specifically the temperature and time, in the development and production of Yesso scallop adductor muscle snacks. The physical, chemical, nutritional, and sensory properties of P-VF under different frying conditions were compared. P-VF demonstrated superior crispiness, supported by microstructural observations, and showed enhanced sensory characteristics in comprehensive evaluations of appearance, taste, aroma, and texture. Furthermore, findings suggest that the quality and shelf life of the product are not compromised by lipid oxidation associated with vacuum low-temperature frying. The results of this study are expected to elicit positive feedback from snack consumers in the Republic of Korea and, more broadly, among consumers in Asia and Europe who favor crispy snack products.

## Figures and Tables

**Table 1:** Symbol, experimental range, and values of the independent variables in the central composite design for optimization of vacuum frying conditions for scallop adductor muscle snacks.

Symbol ^(1)^	Range Level				
	−1.414	−1	0	+1	+1.414
*X*_1_	90.1	93	100	107	109.9
*X*_2_	183	270	480	690	777

^(1)^ *X*_1_: vacuum frying temperature (°C), *X*_2_: vacuum frying time (s).

**Table 2:** Central composite design of independent variables and responses of dependent variables in vacuum frying conditions for scallop adductor muscle snacks.

Coefficients Assessed by	Run No.	Coded Value		Uncoded Value		Dependent Variable		
		*X*_1_	*X*_2_	*X*_1_	*X*_2_	*Y*_1_	*Y*_2_	*Y*_3_
Fractional factorial design (4 points)	1	−1	−1	93.0	270	15.1	424	5.3
	2	+1	−1	107.0	270	12.5	1060	6.0
	3	−1	+1	93.0	690	9.8	1304	6.4
	4	+1	+1	107.0	690	4.2	1581	6.5
Star points (4 points)	5	−1.414	0	90.1	480	13.8	895	5.5
	6	+1.414	0	109.9	480	4.7	1498	7.2
	7	0	−1.414	100.0	183	18.3	369	5.1
	8	0	+1.414	100.0	777	4.5	1511	6.8
Central points (3 points)	9	0	0	100.0	480	5.8	1476	7.5
	10	0	0	100.0	480	5.6	1487	7.4
	11	0	0	100.0	480	5.8	1482	7.5

X1: vacuum frying temperature (°C), X2: vacuum frying time (s), Y1: moisture (g/100 g), Y2: fracturability (g/cm^2^), Y3: sensory chewiness (score).

**Table 3:** Proximate component, energy, and mineral of scallop adductor muscle snacks by frying conditions (C-DF, deep frying; C-AF, air frying; P-VF, vacuum low-temperature frying).

Component		Frying Method		
		C-DF	C-AF	P-VF
Proximate component (g/100 g)	Moisture	35.5 ± 0.1 ^b^	36.0 ± 0.1 ^b^	5.9 ± 0.2 ^a^
	Crude protein	27.9 ± 0.6 ^a^	33.9 ± 0.4 ^b^	45.6 ± 0.2 ^c^
	Crude lipid	12.2 ± 0.4 ^b^	7.9 ± 0.4 ^a^	8.8 ± 1.2 ^a^
	Ash	2.8 ± 0.1 ^a^	2.9 ± 0.1 ^a^	6.5 ± 0.1 ^c^
	Carbohydrate ^(1)^	21.6	19.3	33.2
Energy (kcal/100 g)		312.8	290.7	402.6

^(1)^ Carbohydrate (g/100 g) = 100 − (moisture + crude protein + crude lipid + ash); energy (kal/100 g) = (crude protein × 4.27) + (crude lipid × 9.02) + (carbohydrate × 3.87). Different letters on the data in the column indicate a significant difference at *p* < 0.05.

**Table 4:** Acceptability evaluation of the scallop adductor muscle snacks prototype manufactured using scallop adductor (C-DF, deep frying; C-AF, air frying; P-VF, vacuum low-temperature frying).

Acceptability Evaluation	C-DF	C-AF	P-VF
Appearance	5.9 ± 0.6 ^b^	4.9 ± 0.6 ^a^	8.0 ± 0.7 ^c^
Taste	5.2 ± 0.8 ^a^	6.9 ± 0.7 ^a^	8.2 ± 0.6 ^b^
Flavor	7.0 ± 1.1 ^a^	6.6 ± 0.5 ^a^	6.8 ± 0.6 ^a^
Texture	5.7 ± 0.5 ^a^	5.0 ± 0.9 ^a^	8.3 ± 0.6 ^b^
Overall acceptance	5.5 ± 0.6 ^a^	6.2 ± 0.9 ^a^	7.9 ± 0.7 ^b^

Panel configurations, *n* = 30 (15 males and 15 females). Different letters on the data in the column indicate a significant difference at *p* < 0.05.

**Table 5:** Estimated coefficients of the fitted quadratic polynomial equation for different responses in vacuum frying process optimization of scallop adductor muscle snacks.

	*Y*_1_		*Y*_2_		*Y*_3_	
	Coefficient	*p*-Value	Coefficient	*p*-Value	Coefficient	*p*-Value
Intercept	5.733	0.000	1481.7	0.000	7.467	0.000
*X*_1_	−2.634	0.002	220.7	0.000	0.401	0.012
*X*_2_	−4.140	0.000	377.0	0.000	0.501	0.005
*X*_1_ *X*_1_	1.777	0.017	−136.6	0.000	−0.583	0.005
*X*_2_ *X*_2_	2.852	0.002	−264.8	0.000	−0.783	0.001
*X*_1_ *X*_2_	−0.750	0.265	−89.7	0.005	−0.150	0.353

*X*_1_, vacuum frying temp. (°C); *X*_2_, vacuum frying time (s); *Y*_1_, moisture (g/100 g); *Y*_2_, fracturability (g/cm^2^); *Y*_3_, sensory chewiness (score).

**Table 6:** Optimum frying conditions predicted for preparation in vacuum frying conditions of scallop adductor muscle snacks by MINITAB program.

Dependent Variables	Value		*X*_1_	*X*_2_	
*Y*_1_	Target	5.60	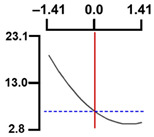	5.60	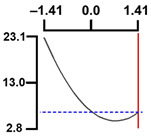
	Coded	0.00		1.41	
	Actual	100.0		777.0	
*Y*_2_	Target	1480	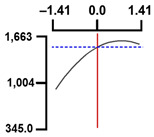	1480	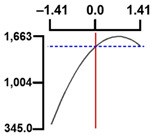
	Coded	0.00		0.00	
	Actual	100.0		480.0	
*Y*_3_	Target	Max	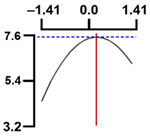	Max	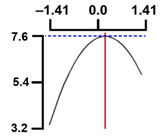
	Coded	0.30		0.30	
	Actual	102.1		543	
Multiple response optimization	Coded		−0.01	0.0	
	Actual		99.9	480	
	Predicted		*Y*_1_: 5.7 g/100 g, *Y*_2_: 1478 g/cm^2^, *Y*_3_: 7.5 score		

*X*_1_, vacuum frying temp. (°C); *X*_2_, vacuum frying time (s); *Y*_1_, moisture (g/100 g); *Y*_2_, fracturability (g/cm^2^); *Y*_3_, sensory chewiness (score).

**Table 7:** Verification of predicted values in vacuum frying conditions in the preparation of scallop adductor muscle snacks.

Dependent Variables	Predicted Values	Experimental Values
Y_1_ (Moisture, g/100 g)	5.7 ^a^	5.6 ± 0.1 ^a^
Y_2_ (Fracturability, g/cm^2^)	1478 ^a^	1470 ± 5.0 ^a^
Y_3_ (Sensory chewiness, score)	7.5 ^a^	7.6 ± 0.2 ^a^

The letter a in the data indicates a significant difference at *p* > 0.05.

## Data Availability

The data presented in this study are available on request from the corresponding author. The data are not publicly available due to privacy restrictions.
